# Comparative mitogenomic analysis of the superfamily Pentatomoidea (Insecta: Hemiptera: Heteroptera) and phylogenetic implications

**DOI:** 10.1186/s12864-015-1679-x

**Published:** 2015-06-16

**Authors:** Ming-Long Yuan, Qi-Lin Zhang, Zhong-Long Guo, Juan Wang, Yu-Ying Shen

**Affiliations:** State Key Laboratory of Grassland Agro-Ecosystems, College of Pastoral Agricultural Science and Technology, Lanzhou University, Lanzhou, Gansu 730020 People’s Republic of China

## Abstract

**Background:**

Insect mitochondrial genomes (mitogenomes) are the most extensively used genetic marker for evolutionary and population genetics studies of insects. The Pentatomoidea superfamily is economically important and the largest superfamily within Pentatomomorpha with over 7,000 species. To better understand the diversity and evolution of pentatomoid species, we sequenced and annotated the mitogenomes of *Eurydema gebleri* and *Rubiconia intermedia*, and present the first comparative analysis of the 11 pentatomoid mitogenomes that have been sequenced to date.

**Results:**

We obtained the complete mitogenome of *Eurydema gebleri* (16,005 bp) and a nearly complete mitogenome of *Rubiconia intermedia* (14,967 bp). Our results show that gene content, gene arrangement, base composition, codon usage, and mitochondrial transcription termination factor sequences are highly conserved in pentatomoid species, especially for species in the same family. Evolutionary rate analyses of protein-coding genes reveal that the highest and lowest rates are found in *atp8* and *cox1* and distinctive evolutionary patterns are significantly correlated with the G + C content of genes. We inferred the secondary structures for two rRNA genes for eleven pentatomoid species, and identify some conserved motifs of RNA structures in Pentatomidea. All tRNA genes in pentatomoid mitogenomes have a canonical cloverleaf secondary structure, except for two tRNAs (*trnS1* and *trnV*) which appear to lack the dihydrouridine arm. Regions that are A + T-rich have several distinct characteristics (e.g. size variation and abundant tandem repeats), and have potential as species or population level molecular markers. Phylogenetic analyses based on mitogenomic data strongly support the monophyly of Pentatomoidea, and the estimated phylogenetic relationships are: (Urostylididae + (Plataspidae + (Pentatomidae + (Cydnidae + (Dinidoridae + Tessaratomidae))))).

**Conclusions:**

This comparative mitogenomic analysis sheds light on the architecture and evolution of mitogenomes in the superfamily Pentatomoidea. Mitogenomes can be effectively used to resolve phylogenetic relationships of pentatomomorphan insects at various taxonomic levels. Sequencing more mitogenomes at various taxonomic levels, particularly from closely related species, will improve the annotation accuracy of mitochondrial genes, as well as greatly enhance our understanding of mitogenomic evolution and phylogenetic relationships in pentatomoids.

**Electronic supplementary material:**

The online version of this article (doi:10.1186/s12864-015-1679-x) contains supplementary material, which is available to authorized users.

## Background

Insect mitochondrial genomes (mitogenomes) are circular double-stranded molecules 15–18 kb in size that usually code for 37 genes: 13 protein-coding genes (PCGs), two ribosomal RNA genes (rRNAs), and 22 transfer RNA genes (tRNAs) [1, 2]. In addition, mitogenomes usually contain a large non-coding region (known as the “A + T-rich” region in insects) that contains essential regulatory elements for transcription and replication [1, 3]. In the past decade, mitogenome sequencing has increased dramatically and these sequences have been extensively used in the study of molecular evolution, phylogenetics, phylogeography and population genetics [2, 4–6].

Pentatomoidea (Hemiptera: Heteroptera: Pentatomomorpha) consists of about 7,000 known species in 15 families, of which Pentatomidae is the largest family containing over 4,700 species in ca. 900 genera [7]. Most species of this superfamily are economically important as agricultural pests, whereas some are used as biological control agents. Gapud [8] used morphological characters to investigate the phylogenetic relationships within Pentatomoidea and found Urostylididae to be the sister group to all remaining pentatomoid species. However, Xu [9] argued that Cydnidae is the earliest diverging group within Pentatomoidea based on morphological data. Grazia *et al*. [10] performed the first phylogenetic analysis within Pentatomoidea based on combined morphological and molecular data, and confirmed that Urostylididae is sister to the other pentatomoid species. Lis *et al*. [11] used two mitochondrial genes (*rrnL* and *rrnS*) to examine the position of Dinidoridae within Pentatomoidea, and identified Dinidoridae as monophyletic and sister to the Tessaratomidae. Analysis of the partial *cox2* sequence, however, did not support a close affinity between Dinidoridae and Tessaratomidae [12]. Currently, the monophyly of Pentatomoidea has been strongly supported in a large number of studies that have been based on morphological and molecular analyses [8, 10, 13–15]. Although considerable research effort has been expended on Pentatomoidea, phylogenetic relationships within the superfamily are still unresolved and controversial [10]. Much of the previous work on pentatomoid relationships has focused mainly on morphological characteristics, and robust phylogeny remains to be explored using mitogenomic data.

To date, only 24 complete or nearly complete mitogenomes have been sequenced for Pentatomomorpha (GenBank, October 10, 2014), of which nine are from Pentatomoidea. The number of sequenced pentatomoid mitogenomes is still quite limited compared to the species-richness of Pentatomoidea and restricts our understanding of evolution in pentatomoid species at the genomic level. In addition, the accurate delimitation of mitochondrial genes may be difficult and error prone when annotated mitogenomes are not available from closely related species [16–18]. Therefore, it is important to sequence more mitogenomes from Pentatomoidea, especially from closely related species, to improve the accuracy of mitochondrial gene and to enhance our understanding of molecular evolution and phylogenetic relationships.

In the present study, we sequenced and annotated the mitogenomes of *Eurydema gebleri* (Strachiini) and *Rubiconia intermedia* (Carpocorini) from the family Pentatomidae. These two species are important alfalfa pests in China. We compared the newly sequenced mitogenomes with nine previously published sequences to check and refine the original annotation of gene boundaries. We provided a detailed comparative analysis of eleven pentatomoid mitogenomes, including nucleotide composition, codon usage, RNA secondary structure, evolutionary pattern among 13 PCGs, and structural elements within the A + T-rich regions. Finally, we evaluated the phylogenetic utility of mitogenomic sequence data at the taxonomic levels of Pentatomomorpha, Pentatomoidea and Pentatomidae.

## Results and discussion

### Genome organization

**Fig. 1:**
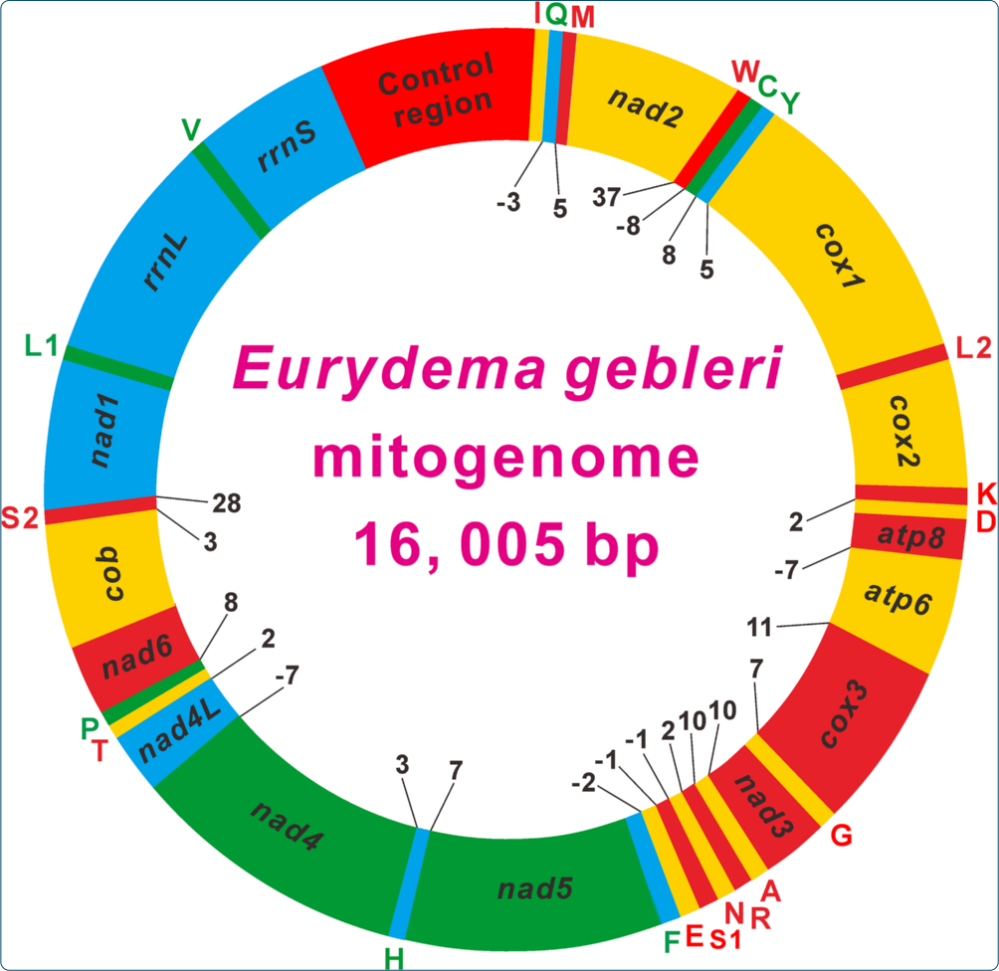
Circular map of the mitochondrial genome of *Eurydema gebleri*. Protein coding and ribosomal genes are shown with standard abbreviations. Genes for tRNAs are abbreviated by a single letter, with S1 = AGN, S2 = UCN, L1 = CUN, and L2 = UUR. Genes coded in the J-strand (clockwise orientation) are red or orange colored. Genes coded in the N-strand (counterclockwise orientation) are green or cyan colored. Numbers at gene junctions indicate the length of small non-coding regions where negative numbers indicate overlap between genes

The complete mitogenome of *E. gebleri* (16,005 bp, GenBank accession KP207595) and the nearly complete mitogenome of *R. intermedia* (14,967 bp, GenBank accession no. KP207596) were obtained (Fig. 1, Additional file 1). The region that we failed to sequence in *R. intermedia* is located between *rrnS* and *nad2*. This area of the mitogenome is known from other insects and contains notable base composition bias, high numbers of tandem repeats, and stable stem-loop structures. These features may result in disruption of PCR and sequencing reactions, as has been reported in other hemipterans [13, 19–21]. The *E. gebleri* mitogenome is a typical circular DNA molecule and its map is shown in Fig. 1.

We found that the boundaries of some genes are incorrectly delimited in the original GenBank sequence files of some pentatomoid species. These errors could have a significant impact on comparative and evolutionary mitogenomic analyses. Therefore, we revised the annotations of these genes, including those encoding for PCGs, rRNAs, tRNAs and A + T-rich regions (Additional file 2). The re-annotated sequences were used for our analyses.

The completely sequenced mitogenomes from Pentatomoidea contain a typical set of 37 mitochondrial genes (13 PCGs, 22 tRNAs and two rRNAs) and an A + T-rich region. Gene arrangement is highly conserved within Pentatomoidea and is identical to the gene order of the putative ancestral insect mitogenome [1, 2]. The mitogenomes of eight completely sequenced pentatomoid species display moderate size variation, ranging from 15,647 bp in *Megacopta cribraria* to 16,889 bp in *Nezara viridula* (Table 1) with most of the size variation being attributed to the variety of non-coding regions, primarily due to length mutations in the A + T-rich regions (Additional file 2). Gene overlaps have been observed at several gene junctions, varying in size from 20 nucleotides in *Dolycoris baccarum* to 30 nucleotides in *Coptosoma bifaria* (Fig. 1, Additional files 1 and 2). The longest overlap (8 bp) exists between *trnW* and *trnC*, whereas two PCG pairs *atp8*/*atp6* and *nad4L*/*nad4* overlap seven nucleotides, in all pentatomoids sequenced so far, except *D. baccarum*. In the *D. baccarum* mitogenome, *atp8* overlaps with *atp6* by only one nucleotide (Additional file 2). The overlap sequences in the three gene junctions are highly conserved among all sequenced pentatomoid mitogenomes (Additional file 3).

**Table 1:** List of the species included in the present study

Infraorder	Superfamily/family	Species	Size (bp)	Accession number	Reference
Cimicomorpha	Miroidea				
	Miridae	*Adelphocoris fasciaticollis*	15,434	NC_023796	[66]
		*Apolygus lucorum*	14,768	NC_023083	[30]
Pentatomomorpha	Aradoidea				
	Aradidae	*Aradacanthia heissi*	15,528	HQ441233	[67]
		*Brachyrhynchus hsiaoi*	15,250	NC_022670	[68]
		*Neuroctenus parus*	15,354	NC_012459	[13]
	Coreoidea				
	Alydidae	*Riptortus pedestris*	17,191	NC_012462	[13]
	Coreidae	*Hydaropsis longirostris*	16,521	NC_012456	[13]
	Rhopalidae	*Aeschyntelus notatus*	14,532	NC_012446^a^	[13]
		*Stictopleurus subviridis*	15,319	NC_012888	[69]
	Lygaeoidea				
	Berytidae	*Yemmalysus parallelus*	15,747	NC_012464	[13]
	Colobathristidae	*Phaenacantha marcida*	14,540	NC_012460^a^	[13]
	Geocoridae	*Geocoris pallidipennis*	14,592	NC_012424^a^	[13]
	Lygaeidae	*Kleidocerys resedae resedae*	14,688	KJ584365	[70]
	Malcidae	*Chauliops fallax*	15,739	NC_020772	[24]
		*Malcus inconspicuus*	15,575	NC_012458	[13]
	Pentatomoidea				
	Cydnidae	*Macroscytus gibbulus*	14,620	EU427338^a^	[13]
	Dinidoridae	*Coridius chinensis*	14,648	JQ739179^a^	[71]
	Pentatomidae	*Dolycoris baccarum*	16,549	NC_020373	[72]
	Pentatomidae	*Eurydema gebleri*	16,005	KP207595	This study
	Pentatomidae	*Halyomorpha halys*	16,518	NC_013272	[73]
	Pentatomidae	*Nezara viridula*	16,889	NC_011755	[13]
	Pentatomidae	*Rubiconia intermedia*	14,967	KP207596^a^	This study
	Plataspidae	*Coptosoma bifaria*	16,179	NC_012449	[13]
	Plataspidae	*Megacopta cribraria*	15,647	NC_015342	Direct Submission
	Tessaratomidae	*Eusthenes cupreus*	16,229	NC_022449	[74]
	Urostylididae	*Urochela quadrinotata*	16,587	NC_020144	[75]
	Pyrrhocoroidea				
	Largidae	*Physopelta gutta*	14,935	NC_012432	[13]
	Pyrrhocoridae	*Dysdercus cingulatus*	16,249	NC_012421	[13]

^a^Incomplete mitochondrial genome

### Nucleotide composition and codon usage

All analyzed pentatomoid mitogenomes are consistently biased towards being AT rich, ranging from 70.4 % in *M. cribraria* to 76.9 % in *N. viridula* (Additional file 4). The lowest A + T content is always found in PCGs, whereas the highest A + T content is found in rRNA-encoding genes (six species) or A + T-rich regions (two species). A + T content also varies for each codon position in PCGs; the third codon position has an A + T content higher than that of the first and second positions (Additional file 4). AT- and GC-skews in pentatomoid mitogenomes are similar to patterns typically found in most insect mitogenomes (Additional file 4), i.e. positive AT-skew and negative GC-skew for the J-strand and the reverse pattern in the N-strand [22]. The PCGs on the J-strand are slightly T- or A-skewed and moderately C-skewed, whereas the N-strand encoded PCGs are all markedly T-skewed and G-skewed. Opposite GC-skew for PCGs between J- and N-strands has also been reported in other insects [23, 24]. There is a marked negative AT-skew in the second codon positions of PCGs on the J-strand, in the three codon positions of PCGs on the N-strand, and in rRNAs. The A + T-rich region, the second and the third positions of PCGs on the J-strand have a markedly negative GC-skew, whereas a significantly biased positive GC-skew is present in the first codon positions of PCGs on both the J- and N-strands, in the third codon positions of PCGs on the N-strand, in rRNAs, and in tRNAs.

Nucleotide composition bias is also reflected in the codon usage pattern. Relative synonymous codon frequencies (RSCU) reveal that four- and two-fold degenerate codon usage is biased to use more As and Ts than Gs and Cs in the third codon positions (Additional file 5). Among 62 amino acids encoding codons in invertebrate mitochondria, some GC-rich codons have never been utilized in some species (Additional file 5). In the *E. gebleri* mitogenome, the four AT-rich codons, TTT-Phe (7.0), TTA-Leu (10.2), ATT-Ile (9.5), and ATA-Met (7.4), are the most frequently used codons. Comparative analyses among other sequenced pentatomoid species also reveal a similar pattern, with values ranging from 25.6 % in *M. cribraria* to 34.9 % in *N. viridula*. Differences in the four most frequently used codons in pentatomoid species seem to be directly linked to the A + T content of all PCGs (R^2^ = 0.96), as has been reported in other insects and chelicerates [16, 25].

**Fig. 2:**
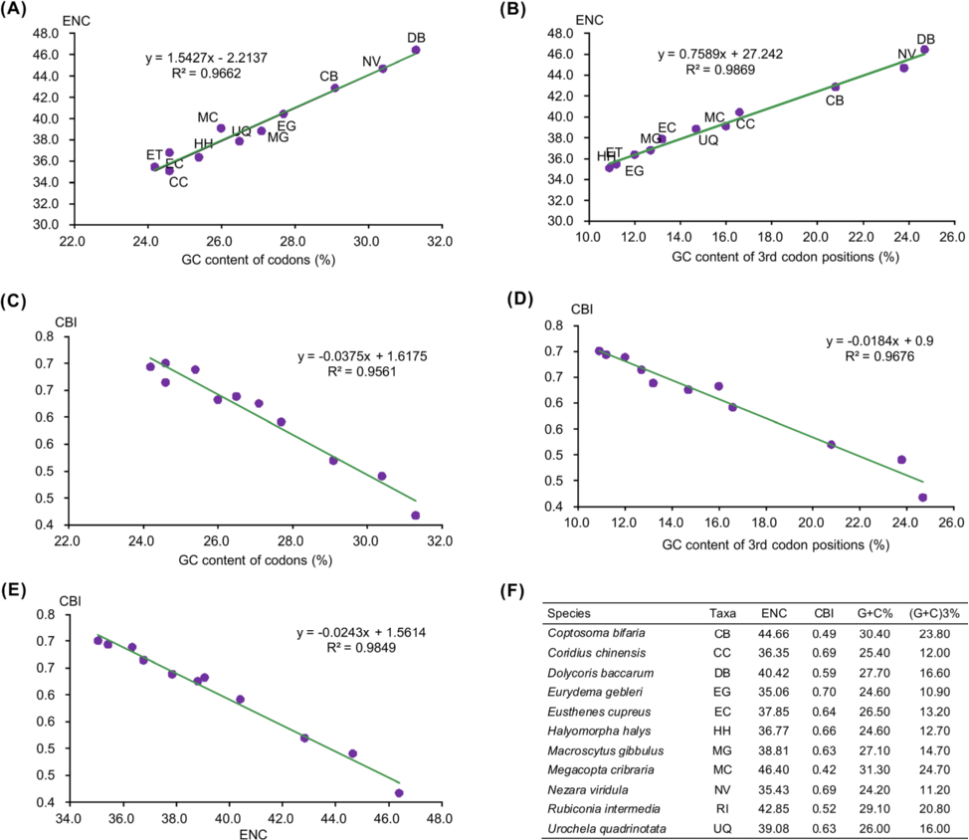
Evaluation of codon bias in the mitochondrial genomes of eleven pentatomoid species. ENC, effective number of codons [63]; CBI, codon bias index [64]; G + C%, G + C content of all codon positions; (G + C)_3_ %, G + C content of the third codon positions

To further investigate the codon usage bias among pentatomoid species, we analyzed the correlations between ENC (effective number of codons), CBI (codon bias index), the G + C content of all codons, and the G + C content of the 3rd codon positions (Fig. 2). We found a positive correlation between ENC and G + C content for all codons (R^2^ = 0.97) (Fig. 2a) and the 3rd codon positions (R^2^ = 0.99) (Fig. 2b). Furthermore, a negative correlation was found between CBI and G + C content for all codons (R2 = 0.96) (Fig. 2c), G + C content of the 3rd codon positions (R2 = 0.97) (Fig. 2d) and ENC (R^2^ = 0.98) (Fig. 2e). These results are consistent with prevailing neutral mutational theories, in that genomic G + C content is the most significant factor in determining codon bias among organisms [26, 27].

### Protein-coding genes

We re-annotated nine PCGs (*nad2*, *cox1*, *atp6*, *nad3*, *nad5*, *nad4L*, *nad6*, *cob* and *nad1*) for nine previously sequenced pentatomoid species (Additional file 2), which changed the size of the genes and the location of the start and stop codons. For *nad2* and *nad6*, the start codons were difficult to determine due to high sequence variation among pentatomoid species. Therefore, we annotated these two genes to have the largest open reading frame, but we did not allow them to overlap with *trnM*, except for *C. bifaria* (Additional file 2).

In pentatomoid mitogenomes, most PCGs initiated with a typical start codon (ATN), but *cox1* always begins with TTG (Additional file 6). In addition, CTG (*nad1*), ATCA (*nad2*), GTG (*nad1* and *nad6*), and TTG (*atp8*, *nad1*, *nad4L*, *nad5* and *nad6*) are also proposed as initiation codons. While most PCGs end with the termination codon TAN, truncated termination codons TA or T are also common (Additional file 6). Truncated stop codons are common in metazoan mitogenomes and may be corrected via post-transcriptional polyadenylation [28, 29].

**Fig. 3:**
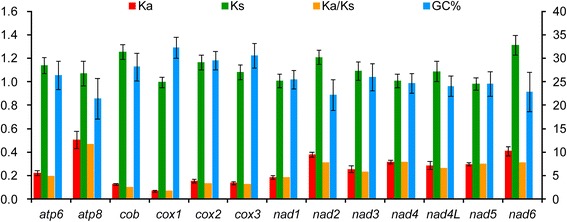
Evolutionary rates of 13 protein-coding genes in the mitochondrial genomes of eleven pentatomoid species. The left Y-axis provides the substitution rate of mitochondrial gene, while the right Y-axis provides the G + C content. Synonymous nucleotide substitutions per synonymous site (Ks) and nonsynonymous nucleotide substitutions per nonsynonymous site (Ka) are calculated using the Kumar method [65]. The standard error estimates are obtained by a bootstrap procedure (1,000 replicates)

In order to investigate the evolutionary patterns among the mitochondrial PCGs in pentatomoid species, the values of Ka, Ks, and Ka/Ks (*ω*) were calculated for each PCG, respectively (Fig. 3). The Ks of *nad6* was the highest, while the values of Ka and *ω* for *atp8* were the highest. At the nucleotide and amino acid levels, four genes (*cox1*, *cox2 cox3*, and *cob*) had the lowest evolutionary rates, suggesting that they are potentially useful barcoding markers. The *ω* values for all PCGs were far lower than one (<0.48), indicating that these genes are evolving under purifying selection. Therefore, all mitochondrial PCGs could be used to investigate phylogenetic relationships within Pentatomoidea. Furthermore, a negative correlation has been found between *ω* and the G + C content of each PCG (R^2^ = 0.89), indicating that variation in G + C content probably causes the different evolutionary patterns among genes.

### Transfer RNA genes

**Fig. 4:**
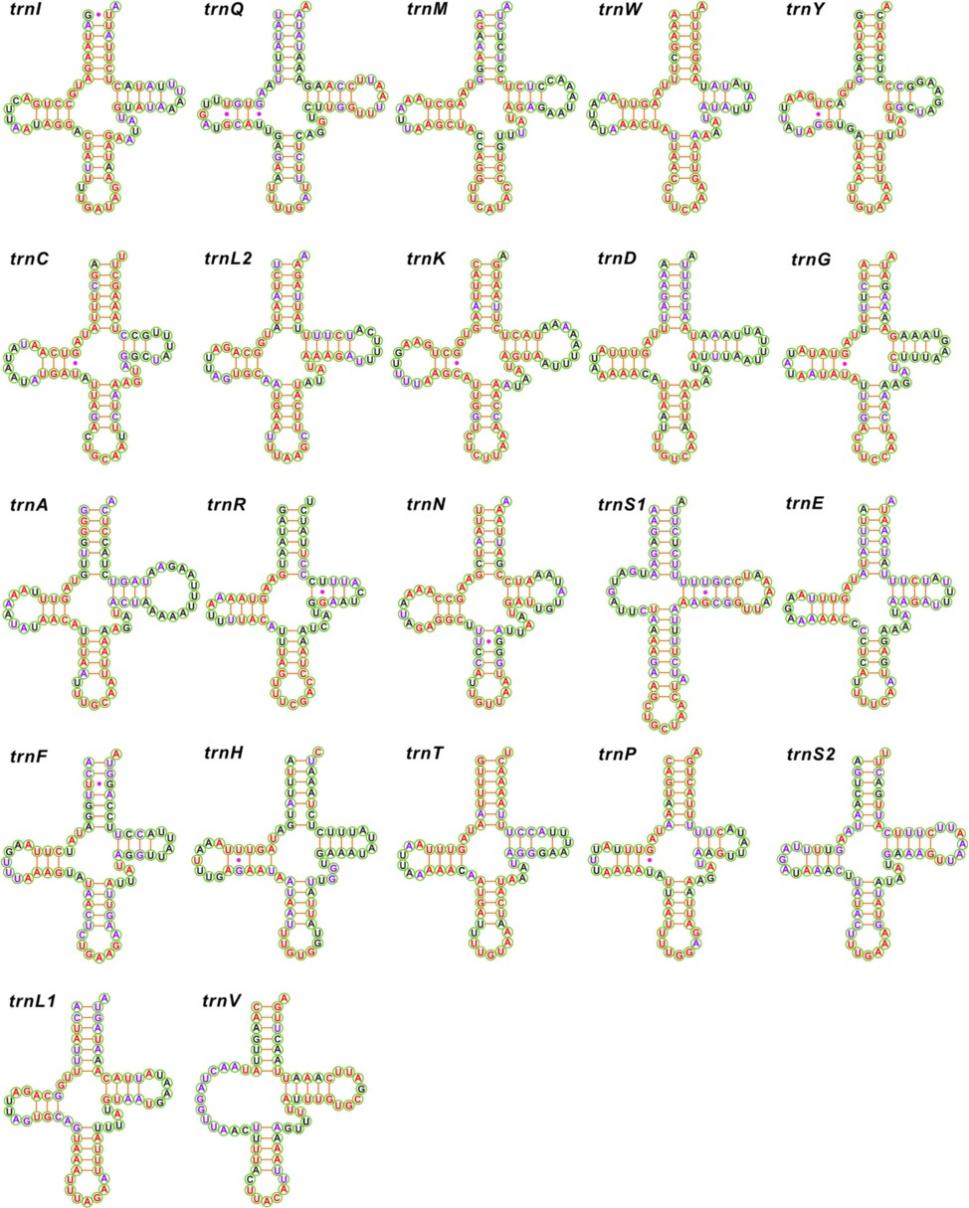
Putative secondary structures of the 22 tRNA genes identified in the mitochondrial genome of *Eurydema gebleri*. All tRNA genes are shown in the order of occurrence in the mitochondrial genome starting from *trnI*. The nucleotides showing 100 % identity within Pentatomoidea and Pentatomidae are marked with red and purple colors, respectively. Bars indicate Watson–Crick base pairings, and dots between G and U pairs mark canonical base pairings in tRNA

We re-annotated six tRNAs, *trnG* (*Halyomorpha halys*), *trnA* (*M. cribraria*), *trnR* (*C. bifaria* and *Urochelas quadrinotata*), *trnF* and *trnH* (*H. halys* and *M. cribraria*), and *trnS1* (*Coridius chinensis*, *Eusthenes cupreus*, *H. halys*, *Macroscytus gibbulus*, and *N. viridula*) (Additional file 2). The secondary structures of these six tRNAs are well conserved, though their sequences are the most variable among 22 tRNAs (Additional file 7). All 22 tRNAs typical of metazoan mitogenomes are present in most pentatomoid mitogenomes we assessed, with an average size ranging from 64 ± 0.8 bp to 73 ± 1.3 bp (Additional file 2). In the *E. gebleri* mitogenome, twenty tRNAs possess a canonical cloverleaf secondary structure composed of four arms with conserved size, whereas the remaining two tRNAs (*trnS1* and *trnV*) appear to lack the dihydrouridine (DHU) arm (Fig. 4). Similar structures have also been observed in other pentatomoid mitochondrial sequences and conserved nucleotides for each tRNA within Pentatomoidea and Pentatomidae are given in Fig. 4. The sequences and structures of anticodon arms, aminoacyl acceptor stems, and DHU stems are highly conversed, especially within Pentatomidae, and most of the nucleotide variation is restricted to DHU and pseudouridine (TψC) loops and variable arms, with obvious indel polymorphisms (Fig. 4).

The loss of the DHU arm in *trnS1* (AGN) has been considered a typical feature of metazoan mitogenomes [3]. However, we found that *trnS1* in eleven pentatomoid mitogenomes possesses an unusual anticodon stem (9 bp vs. the normal 5 bp) with a bulged nucleotide (Fig. 4, Additional file 7). This is an unusual phenomenon, but has also been observed in the DHU arm in other true bugs [19, 24, 30–32]. In *U. quadrinotata* and *E. cupreus*, *trnV* has an extremely short (only 1 bp) DHU stem, suggesting that this tRNA may have lost the DHU arm, as has been observed in other pentatomoid species (Fig. 4, Additional file 7). In insect mitogenomes, apart for the loss of the DHU arm in *trnS1*, truncated tRNA structures have only been found in cecidomyiids [2], where each tRNA gene has lost the entire TψC arm and the 3′ end of the aminoacyl acceptor stem [33]. The loss of the DHU arm of tRNAs is an uncommon occurrence, but this phenomenon has also been reported for some mitochondrial tRNAs from chelicerates [16]. Although functional tRNAs that lack a DHU arm have not been found in insects, it has been reported that in the nematode *Ascaris suumthe* tRNAs may lack either the DHU or the TψC arm and retain functionality [34].

### Ribosomal RNA genes

As in other insect mitogenomes, pentatomoid mitogenomes have two genes encoding the large and small rRNA subunits (*rrnL* and *rrnS*) that are located at a conserved position between *trnL1* (CUN) and *trnV*, and between *trnV* and the A + T-rich region, respectively (Fig. 1, Additional files 1 and 2). We re-annotated the boundaries of *rrnS* (*U. quadrinotata*) and *rrnL* (*C. chinensis* and *H. halys*) (Additional file 2). The length of *rrnL* varies from 1,247 bp in *U. quadrinotata* to 1,309 bp in *M. gibbulus*, whereas the largest and smallest *rrnS* genes are 814 bp in *N. viridula* and 779 bp in *M. gibbulus*. Therefore, there was not substantial size variation between rRNAs within the family Pentatomidae (1,278 ± 8 bp in *rrnL* and 807 ± 9 bp in *rrnS*).

**Fig. 5:**
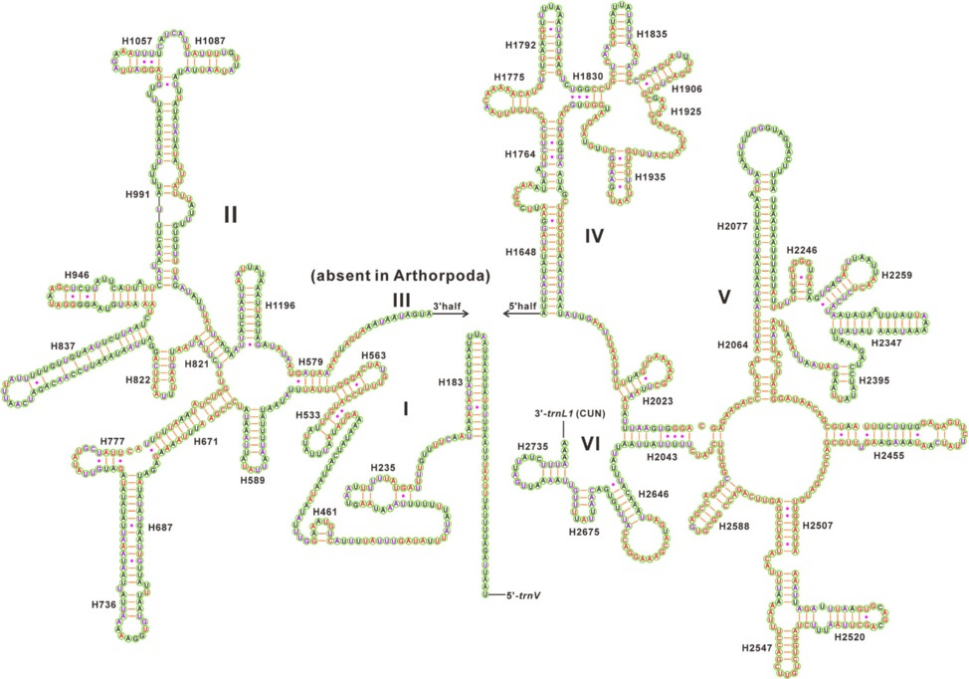
Predicted secondary structure for the *rrnL* in the mitochondrial genome of *Eurydema gebleri*. The nucleotides showing 100 % identity within Pentatomoidea and Pentatomidae are marked with red and purple colors, respectively. Inferred Watson-Crick bonds are illustrated by lines, whereas GU bonds are illustrated by dots

**Fig. 6:**
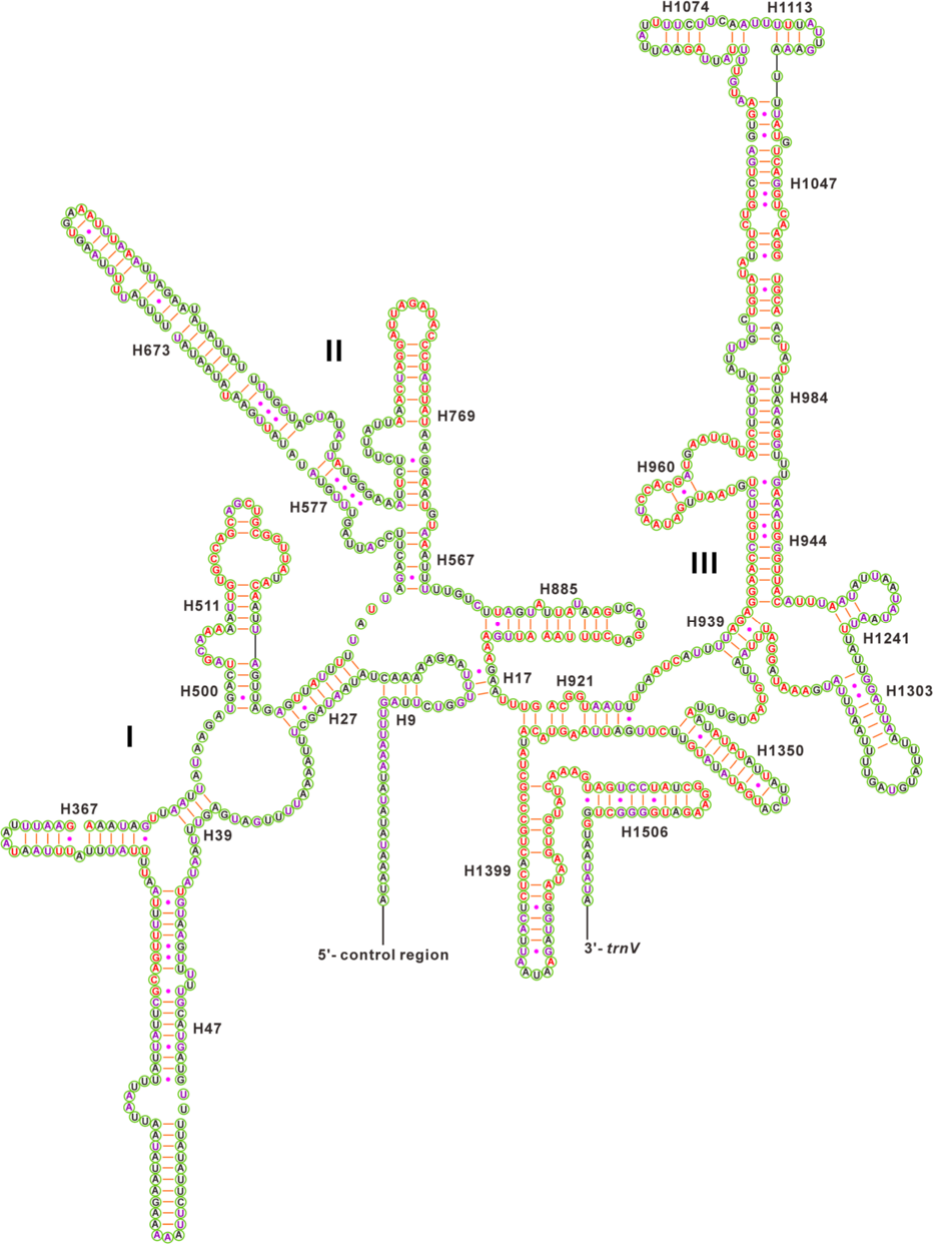
Predicted secondary structure for the *rrnS* in the mitochondrial genome of *Eurydema gebleri*. The nucleotides showing 100 % identity within Pentatomoidea and Pentatomidae are marked with red and purple colors, respectively. Inferred Watson-Crick bonds are illustrated by lines, whereas GU bonds are illustrated by dots

In this study the secondary structures of the rRNAs of *E. gebleri* were constructed following the models proposed for other insects, and compared to other pentatomoid species. The secondary structures of *rrnL* and *rrnS* inferred for *E. gebleri* have similar stem-loop structures as those proposed for *D. melanogaster* [35], *A. mellifera* [36], *M. sexta* [37] and other hemipterans (e.g. *Chauliops fallax* [24], *Stenopirates* sp. [32] and *Cavariella salicicola* [20]). The secondary structure of *rrnL* consists of six structural domains (domain III is absent in arthropods) and 44 helices (Fig. 5), whereas the secondary structure of *rrnS* contains three domains and 26 helices (Fig. 6).

In *rrnL*, domains IV and V are more conserved within Pentatomoidea than domains I, II, and VI. Six helices (H563, H1775, H2064, H2507, H2547 and H2588) of *rrnL* are highly conserved with 0–2 nucleotide substitutions. Some helices (H183, H687, H736, H837, H991, H2077 and H2347) are highly variable in terms of their sequence and secondary structure, as has been frequently observed in other insects [20, 24, 32].

Compared to the 5′-end, the 3′-end of *rrnS* is structurally more conserved within Pentatomoidea, especially in the helices H921-960, H1047 and H1399. The helix H47 is highly variable between different insects; in fact no consistent structure has been found for this region [37]. The estimated secondary structure of H47 in *E. gebleri* consists of a long stem and a short terminal loop, as is also found in *Stenopirates* sp. [32] and *C. fallax* [24]. The other helices (H1047, H1068, H1074 and H1113) are highly variable and Mfold estimates several likely secondary structures due to the sequence’s high A + T bias and several non-canonical base pairs [20, 24, 32, 36, 37]. However, the helix H1047 is well conserved in terms of its sequence and structure within Pentatomoidea (Fig. 6). Although the helix H1068 has been found in some insects [24, 36–38], this helix seems to be absent in the *rrnS* of *E. gebleri*, as found in *Stenopirates* sp. [32] and *C. salicicola* [20]. In most insects, the helix H673 usually forms a short paired stem with a large loop, but this helix in *E. gebleri*, H673 forms a long stem and a very small loop. Comparison of this helix among pentatomoid species shows that its nucleotide sequences are highly variable, and thus this structure might be taxon-specific.

### Non-coding regions

Pentatomoid mitogenomes are highly economized in genome size, just as in other animals, and they contain only a few intergenic spacers, most of which are shorter than ten nucleotides (Additional files 1 and 2). The longest intergenic spacer sequence we found is located between *trnS2* (UCN) and *nad1* in all pentatomoid species, except for *M. cribraria* and *E. gebleri* (Additional files 1 and 2). This intergenic spacer is also present in other insect orders [37] and these sequences have been shown to be the binding site of a transcription termination factor (DmTTF) [39]. All of the *trnS2*-*nad1* intergenic spacer sequences observed in pentatomoid mitogenomes are highly conserved and have a conserved motif with significant similarity to the DmTTF binding site in *D. melanogaster* (Additional file 8).

**Fig. 7:**
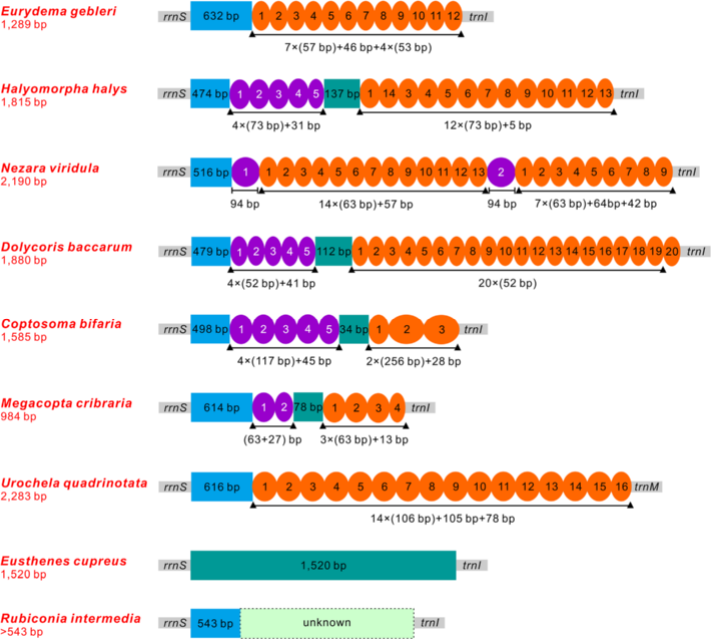
Organization of the A + T-rich region in pentatomoid mitochondrial genomes. The location and copy number of tandem repeats are shown by colored oval with Arabic numerals inside. Non-repeat regions are indicated by colored box with sequence size inside

The largest non-coding region (which is an A + T-rich region) in the eight pentatomoid mitogenomes is located at a conserved position as in most insects (Fig. 1, Additional files 1 and 2). A + T-rich regions in the eight pentatomoid mitogenomes have varying lengths, ranging from 984 bp in *M. cribraria* to 2,283 bp in *U. quadrinotata* (Additional files 1 and 2). A comparison of structures in A + T-rich regions among pentatomoid mitogenomes is shown in Fig. 7. With the exception of *E. cupreus*, A + T-rich regions in the other seven species include large tandem repeats present as two or more copies. In addition, *rrnS* borders a large non-repeat region (474–632 bp) that is present in all of the eight species (Fig. 7) and its G + C content (27.5–34.7 %) is higher than the average G + C content of the whole genome. In most pentatomoid species, tandem repeat sequences contain two types of repeat units and are interrupted by a non-coding region. Although pentatomoid A + T-rich regions form several stem-loop structures, no conserved functional motifs could be identified. Overall, pentatomoid A + T-rich regions show distinct sequence and structural characteristics, such as varying sizes and differentiated tandem repetitions. These characteristics may be taxon-specific and can potentially be used as genetic markers for evolutionary and population genetic studies of pentatomoid species.

### Phylogenetic analysis

**Fig. 8:**
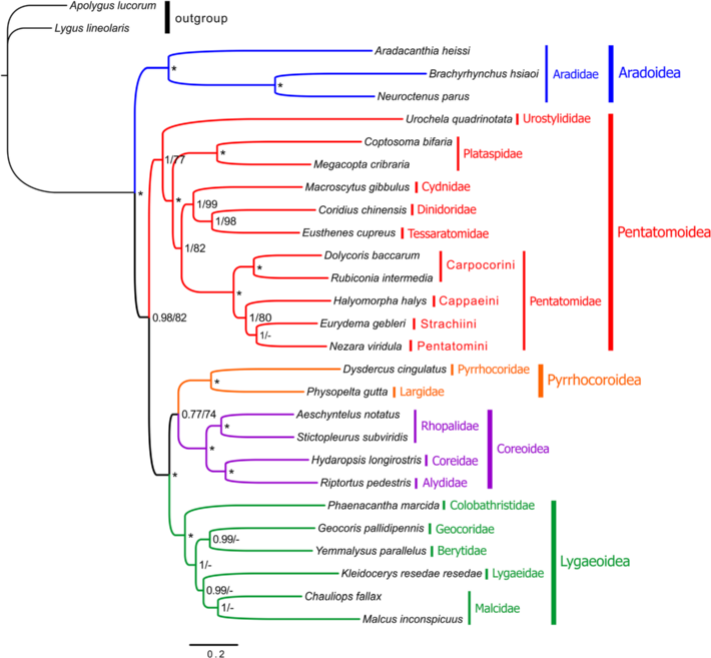
Phylogenetic relationships among five Pentatomomorpha superfamilies based on the concatenated nucleotide sequences of 13 mitochondrial protein-coding genes (the dataset P123). Numbers on branches are Bayesian posterior probabilities (PP, left) and Bootstrap values (BS, right). Asterisk (*) indicates PP = 1.0 and BS = 100

We performed phylogenetic analyses with four datasets (P12, P123, P12LRT, and P123RT) and two inference methods (BI and ML) (Fig. 8, Additional files 9, 10 and 11). We found that tree topologies were sensitive to different datasets and inference methods, and support values were higher in the BI tree than in the ML tree using the same dataset. Although sequences of two rRNAs and 22 tRNAs have been used in previous hemipteran phylogenetic studies (e.g., [21, 40, 41]), our analyses with RNAs (P12LRT and P123RT) do not improve branch support in ML and BI trees, but actually reduce support for some branches. Phylogenetic analyses that included RNA genes did not recover a sister-group relationship between the Aradoidea and the Trichophora. In contrast, analyses based on two PCG datasets (P12 and P123) strongly support the sister-group relationship of Aradoidea and the Trichophora, and place Pentatomoidea as sister to the remainder of the Trichophora, as previous analyses based on the morphological and molecular data have indicated [10, 13, 14, 40, 42–45]. Substitution saturation can have negative effects on phylogenetic reconstructions, but the exclusion of saturated sites (i.e., the third codon positions and *rrnS*) did not improve resolution in our analyses (Additional files 9 and 11). These results indicate that RNA data might be unsuitable for reconstructing the organismal evolutionary relationships within Pentatomomorpha.

All analyses consistently recover the five superfamilies (Aradoidea, Pentatomoidea, Pyrrhocoroidea, Lygaeoidea, and Coreoidea) with high support (Fig. 8, Additional files 9, 10 and 11). The clades have previously been established in Pentatomomorpha as monophyletic groups. Incongruent phylogenetic relationships within Eutrichophora (Pyrrhocoroidea, Lygaeoidea, and Coreoidea) have been frequently observed in other molecular studies [13, 40, 43, 46]. Pyrrhocoroidea and Coreoidea are consistently recovered as sister, except in a BI analysis of PCG123RT, and this is congruent with traditional taxonomic hypotheses based on morphology [14] and other molecular phylogenetic studies [24, 43, 46]. However, this result is different from the results of [13] and [40] using mitogenomic data, where Lygaeoidea and Coreoidea are sister. Within Pentatomoidea, all analyses strongly support a relationship of (Urostylididae + (Plataspidae + (Pentatomidae + (Cydnidae + (Dinidoridae + Tessaratomidae))))), which is consistent with previous studies based on morphological and molecular data [8, 10, 11, 46]. Furthermore, the monophyly of Pentatomidae is also strongly supported in all analyses, and a phylogenetic relationship of (Carpocorini, (Cappaeini, (Strachiini + Pentatomini))) within Pentatominae is recognized in most analyses (Fig. 8, Additional files 9, 10 and 11).

Although some conflicting results are observed from different datasets and inference methods, our results largely agree with the traditional morphological classification and recent molecular studies, suggesting that mitogenome sequences are promising genetic markers for resolving phylogenetic relationships at various taxonomic levels of Pentatomomorpha. Further researches with denser taxon sampling and molecular markers from across the nuclear genome, as well as appropriate phylogenetic inference methods are needed to provide more precise estimates for phylogenetic relationships within Pentatomomorpha and Heteroptera.

## Conclusions

In this study we sequenced and annotated the complete mitogenome of *Eurydema gebleri* and the nearly complete mitogenome of *Rubiconia intermedia*. We present the first comparative analysis of eleven pentatomoid mitogenomes and our results show that gene content, gene arrangement, base composition, codon usage, RNA structure, and mitochondrial transcription termination factor sequences are highly conserved in pentatomoids, especially among closely-related species. Although most tRNAs have a canonical cloverleaf secondary structure, abnormal tRNAs are also present in pentatomoid mitogenomes. Phylogenetic relationships within Pentatomomorpha based on mitogenomic data are consistent with the traditional morphological classification, suggesting that mitogenome sequences are useful for resolving phylogenetic relationships at various taxonomic levels. Sequencing more mitogenomes representing various taxonomic levels, particularly from closely related species, will not only improve the accuracy of annotations for mitochondrial genes, but will also greatly improve our understanding of mitogenomic evolution and phylogenetic relationships in pentatomoids.

## Methods

### Samples and DNA extraction

Adult specimens of *Eurydema gebleri* and *Rubiconia intermedia* were collected from an alfalfa field in Xiaguanying Town (Yuzhong County, Gansu Province, China) in July, 2013 and from Shishe Town (Xifeng District, Qingyang City, Gansu Province) in August, 2013. Samples and voucher specimens are deposited in the State Key Laboratory of Grassland Agro-Ecosystems, College of Pastoral Agricultural Science and Technology, Lanzhou University, Lanzhou, China. All specimens were initially preserved in 100 % ethanol in the field, and transferred to −20 °C until used for DNA extraction. For each species, total genomic DNA was extracted from the thorax muscle of a single specimen using a DNeasy Tissue Kit (Qiagen) according to the manufacturer’s protocols.

### PCR Amplification, cloning and sequencing

The mitogenome sequences of *E. gebleri* and *R. intermedia* were amplified in overlapping fragments by a set of universal insect mitochondrial primers [4] and species-specific primers designed from sequenced fragments. All primers used in this study are provided in Additional file 12. PCR reactions were performed with TaKaRa LA Taq under the following conditions: 2 min initial denaturation at 92 °C, followed by 35 cycles of 10 s at 92 °C, 1 min at 48-55 °C, and 1–4 min at 68 °C, and a final elongation for 20 min at 68 °C. All PCR products were electrophoresed on a 1.5 % agarose gel, purified, and then directly sequenced or cloned into the pEASY-T1 vector (TransGen Biotech, Beijing, China). All fragments were sequenced in both directions on an ABI3730 automated sequencer (Applied Biosystems).

### Annotation and bioinformatics analysis

Sequence files were proof read and assembled into contigs with BioEdit 7.0.9.0 [47]. PCGs were identified by ORF Finder implemented through the NCBI website using invertebrate mitochondrial genetic codes. To ensure the accuracy of gene boundaries, all PCG sequences were aligned with mitochondrial sequences of other true bugs using Muscle as implemented in MEGA 6.06 [48]. The ends of rRNA genes are difficult to precisely determine by DNA sequencing alone, so they are assumed to extend to the boundaries of flanking genes [49, 50]. Most of the tRNAs were predicted by their cloverleaf secondary structure using tRNAscan-SE 1.21 [51]. However, some tRNAs that were not detected by tRNAscan-SE were determined through sequence similarity to tRNAs of other true bugs.

Nucleotide composition and codon usage were analyzed with MEGA 6.06 [48]. The number of synonymous substitutions per synonymous site (Ks), the number of nonsynonymous substitutions per nonsynonymous site (Ka), the effective number of codons (ENC) and the codon bias index (CBI) for each PCG were determined with DnaSP 5.0 [52]. Strand asymmetry was calculated using the formulas: AT-skew = [A-T]/[A + T] and GC-skew = [G-C]/[G + C] [53]. The tandem repeats of the A + T-rich region were identified by the tandem repeats finder online server (http://tandem.bu.edu/trf/trf.html) [54] and the stem-loop structure of the A + T-rich region was inferred by the Mfold Web Server (http://mfold.rna.albany.edu/) [55].

### Estimates of rRNA secondary structure

The secondary structure of the large and small subunits of rRNAs (*rrnL* and *rrnS*) were predicted following the models proposed for *Drosophila melanogaster* (Diptera: Drosophilidae) [35], *Apis mellifera* (Hymenoptera: Apidae) [36], *Manduca sexta* (Lepidoptera: Sphingidae) [37], and other hemipterans [20, 24, 32]). Helix numbering follows the convention established at the CRW site [35]. Regions lacking significant homology and other non-coding regions were folded using the Mfold Web Server [55].

### Phylogenetic analysis

Twenty-six Pentatomomorpha species with complete or nearly complete mitogenomes were used in phylogenetic analyses, representing five superfamilies and seventeen families. Two species of Cimicomorpha, *Adelphocoris fasciaticollis* and *Apolygus lucorum*, were used as outgroups. Details of the species used in this study are listed in Table 1.

The complete sequences of 13 PCGs (excluding stop codons), two rRNAs and 22 tRNAs were used for phylogenetic analyses. Each PCG was aligned individually with codon-based multiple alignments using MAFFT as implemented in the TranslatorX online server [56]. Gaps and ambiguous sites were removed from the protein alignment before back-translation to nucleotides using default settings in GBlocks within the TranslatorX. The rRNAs were aligned with MAFFT (http://mafft.cbrc.jp/alignment/server/) using the Q-INS-i method [57], which has been shown to be more accurate than other programs because it optimizes the alignment in accordance with the secondary structures of rRNA. Each tRNA was aligned using Muscle implemented in MEGA 6.06 [48], and the resulting alignments of tRNA were carefully adjusted by eye according to the secondary structures. Ambiguous positions and divergent regions in the alignment of RNA genes were removed using the GBlocks Server (http://molevol.cmima.csic.es/castresana/Gblocks_server.html), allowing gap positions within the final blocks. Alignments of individual genes were then concatenated as a combined matrix. To determine if sequence saturation exists in our alignments, transitions and transversions were plotted against genetic distance estimated with a GTR model of nucleotide evolution, as implemented in DAMBE 5.3.74 [58]. Saturation plots revealed substantial substitution saturation in the third codon positions of 13 PCGs and *rrnS* (Additional file 13). Therefore, phylogenetic analyses were performed with four datasets: (1) first and second codon positions of PCGs (P12); (2) all codon positions of PCGs (P123); (3) P12, *rrnL*, and 22 tRNAs (P12LRT) and (4) P123, two rRNAs, and 22 tRNAs (P123RT).

We used PartitionFinder 1.1.1 [59] to determine the best partitioning schemes and corresponding nucleotide substitution models. We predefined data blocks by genes and codons, e.g., 26 partitions for P12, 39 partitions for P123, 28 partitions for P12LRT, and 42 partitions for P123RT. We used the Bayesian information criterion (BIC) and the “greedy” algorithm with branch lengths estimated as “unlinked” to search for the best-fit scheme (Additional file 14). The best-fit partitioning schemes proposed by PartitionFinder were used in all subsequent phylogenetic analyses.

Phylogenetic analyses were conducted with ML and BI methods available on the CIPRES Science Gateway 3.3 [60]. ML analyses were performed with RAxML-HPC2 on XSEDE 8.0.24 [61] using the GTRGAMMAI model, and node confidence was assessed with 1,000 bootstrap replicates (BS). Bayesian analyses were carried out using MrBayes 3.2.2 [62] on XSEDE. Two independent runs with four chains (three heated and one cold) were conducted simultaneously for 1 × 10^7^ generations. Each set was sampled every 100 generations. Stationarity was considered to be reached when the ESS (estimated sample size) value was above 100 and the PSRF (potential scale reduction factor) approached 1.0 as suggested by the authors of MrBayes 3.2.2 [62]. The first 25 % of samples were discarded as burn-in and the remaining trees were used to calculate posterior probabilities (PP) in a 50 % majority-rule consensus tree.

### Availability of supporting data

Phylogenetic data (alignments and phylogenetic trees) have been deposited in the TreeBASE (http://purl.org/phylo/treebase/phylows/study/TB2:S17642).

## Additional files

Additional file 1:
**Annotation and organization of the mitochondrial genomes of** ***Eurydema gebleri*** **and** ***Rubiconia intermedia*** **.**
Additional file 2:
**Re-annotation and comparison of mitochondrial genome organizations of nine previously sequenced pentatomoid species.**
Additional file 3:
**Alignment of the three longest gene overlaps among the mitochondrial genomes of eleven pentatomoid species.**
Additional file 4:
**Nucleotide composition of mitochondrial genomes of eleven pentatomoid species.**
Additional file 5:
**Codon usage for the 13 mitochondrial protein-coding genes of eleven pentatomoid species.**
Additional file 6:
**Start and stop codons of mitochondrial protein-coding genes of eleven pentatomoid species.**
Additional file 7:
**Sequence alignment of seven tRNA genes among eleven pentatomoid species.**
Additional file 8:
**Sequence alignments of a large intergenic spacer (between** ***trnS2 and nad1*** **) between eleven pentatomoid species and** ***Drosophila melanogaster*** **.**
Additional file 9:
**Bayesian (A) and maximum likelihood (B) phylogeny of Pentatomomorpha inferred from the dataset P12.**
Additional file 10:
**Bayesian (A) and maximum likelihood (B) phylogeny of Pentatomomorpha inferred from the dataset P123RT.**
Additional file 11:
**Bayesian (A) and maximum likelihood (B) phylogeny of Pentatomomorpha inferred from the dataset P12LRT.**
Additional file 12:
**Primers used in this study.**
Additional file 13:
**Saturation plots for 13 protein-coding genes (PCGs), 2 rRNA genes (** ***rrnL*** **and** ***rrnS*** **) and 22 tRNA genes.**
Additional file 14:
**The best partitioning scheme selected by PartitionFinder for different datasets.**

## Abbreviations

PCGs: Protein-coding genes
*atp6* and *atp8*: Genes for the ATPase subunits 6 and 8
*cox1*-*cox3*: Genes for cytochrome C oxidase subunits I-III
*cob*: A gene for apocytochrome b
*nad1*-*nad6* and *nad4L*: Genes for NADH dehydrogenase subunits 1–6 and 4 L
*rrnL*: Large (16S) rRNA subunit (gene)
*rrnS*: Small (12S) rRNA subunit (gene)
*trnX* (where X is replaced by one letter amino acid code of the corresponding amino acid): Transfer RNA
