# Supplementary figures and images for: Comparative mitogenomic analysis of the superfamily Pentatomoidea (Insecta: Hemiptera: Heteroptera) and phylogenetic implications
Source: BMC Genomics. 2015 Jun 16;16(1):460. doi: 10.1186/s12864-015-1679-x (PMC4469028; doi:10.1186/s12864-015-1679-x)

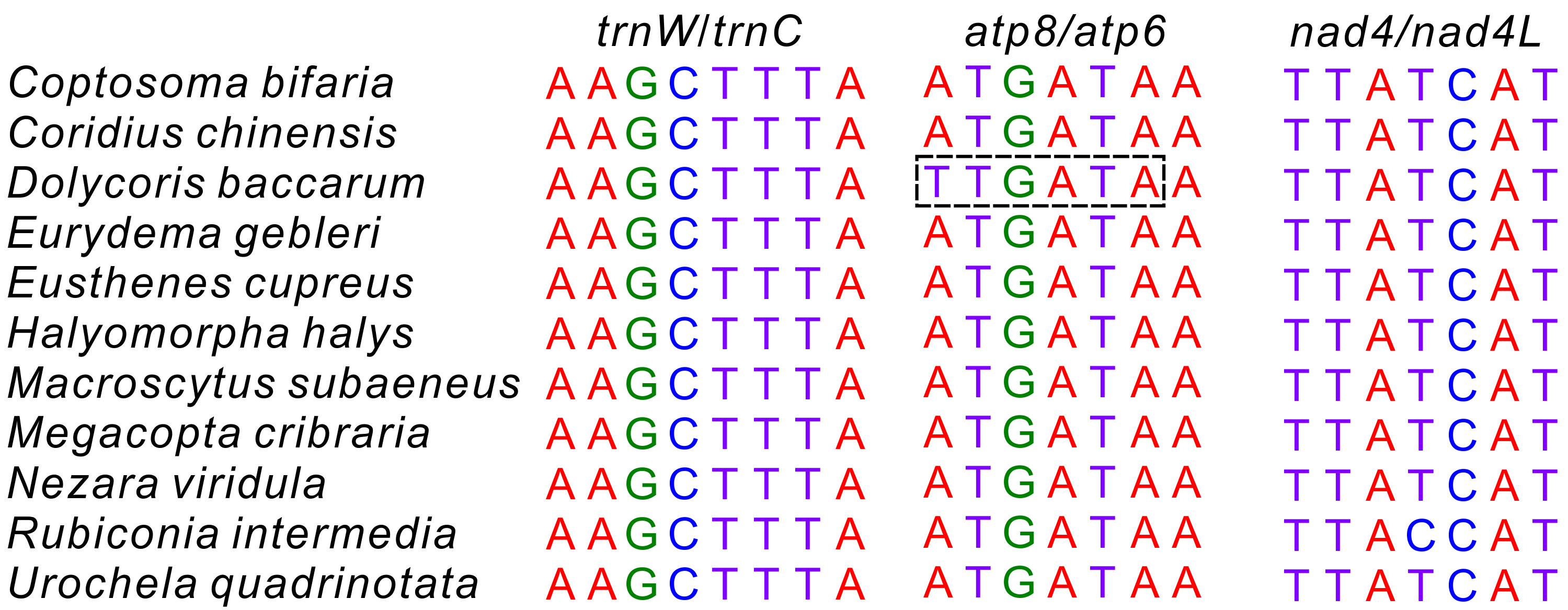

Supplement: Additional file 3: — Alignment of the three longest gene overlaps among the mitochondrial genomes of eleven pentatomoid species. [file 12864_2015_1679_MOESM3_ESM.tiff]

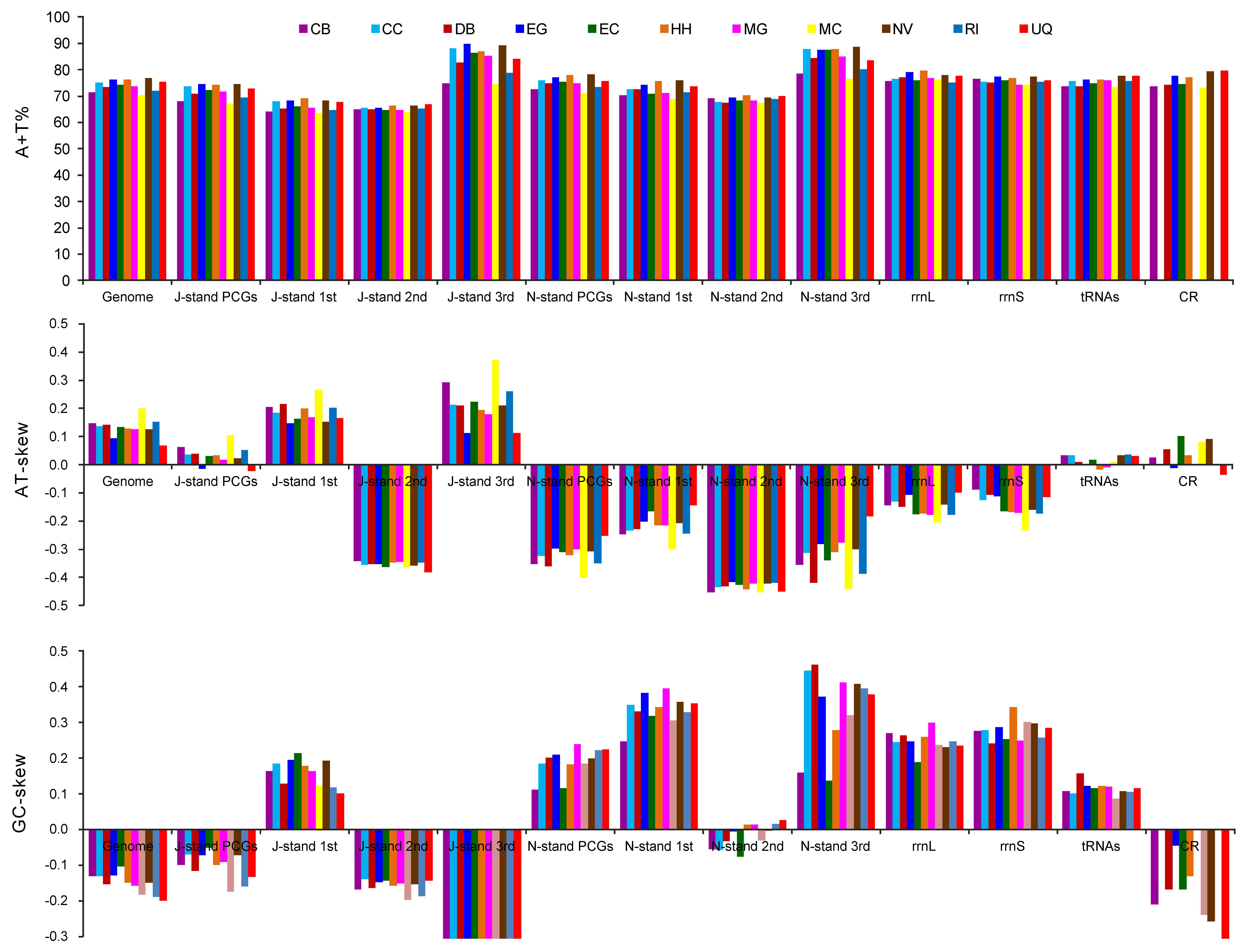

Supplement: Additional file 4: — Nucleotide composition of mitochondrial genomes of eleven pentatomoid species. [file 12864_2015_1679_MOESM4_ESM.tiff]

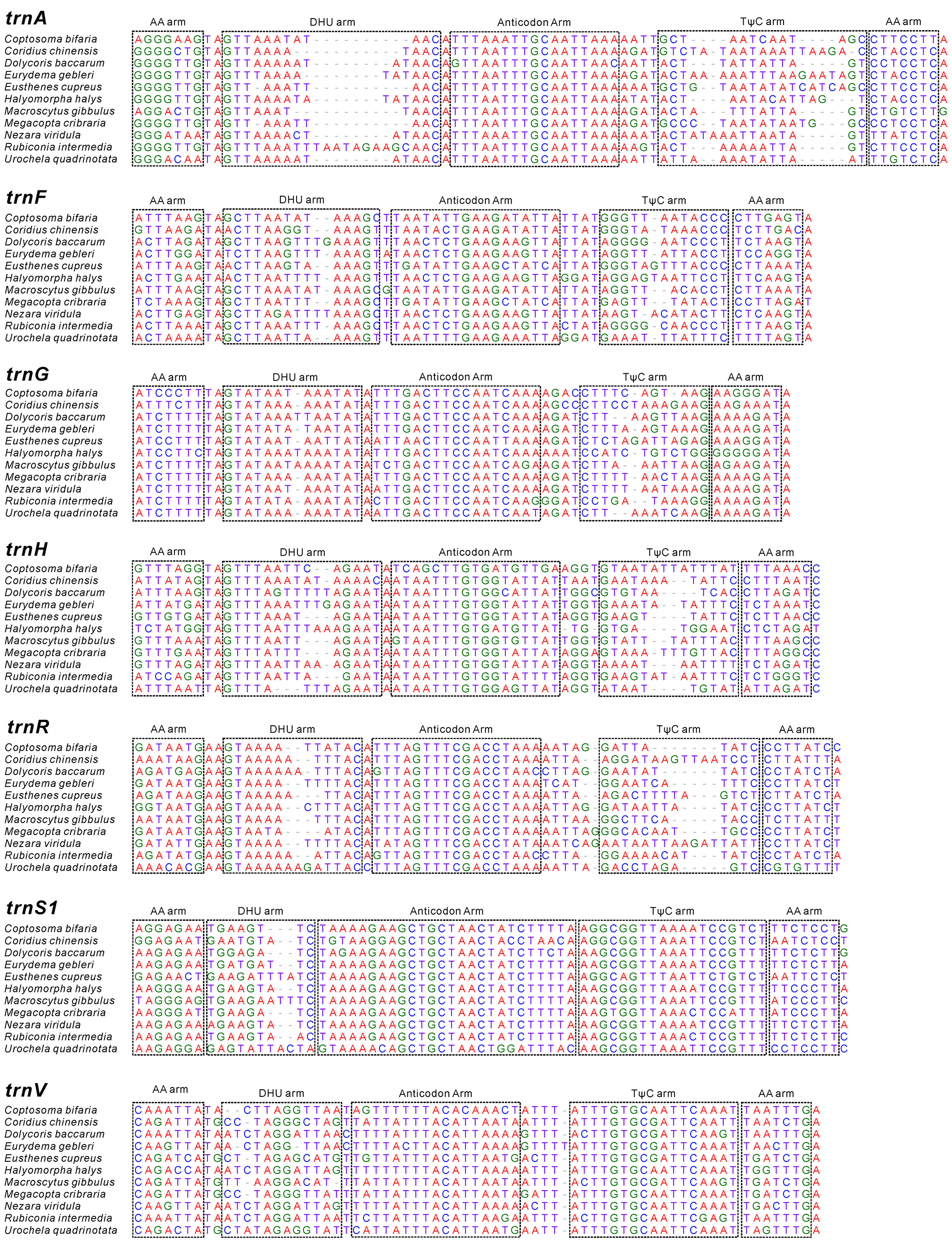

Supplement: Additional file 7: — Sequence alignment of seven tRNA genes among eleven pentatomoid species. [file 12864_2015_1679_MOESM7_ESM.tiff]

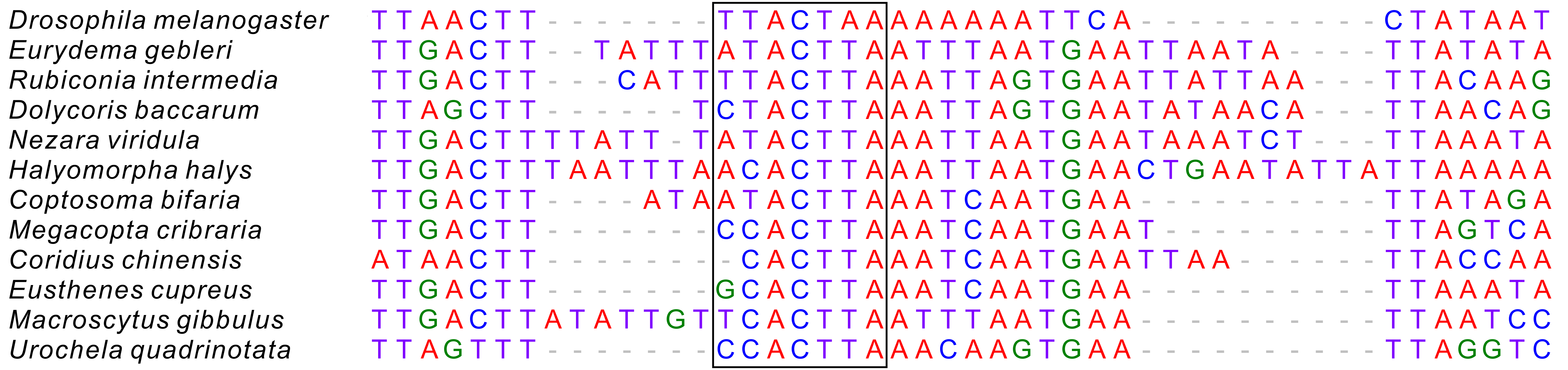

Supplement: Additional file 8: — Sequence alignments of a large intergenic spacer (between trnS2 and nad1 ) between eleven pentatomoid species and Drosophila melanogaster . [file 12864_2015_1679_MOESM8_ESM.tiff]

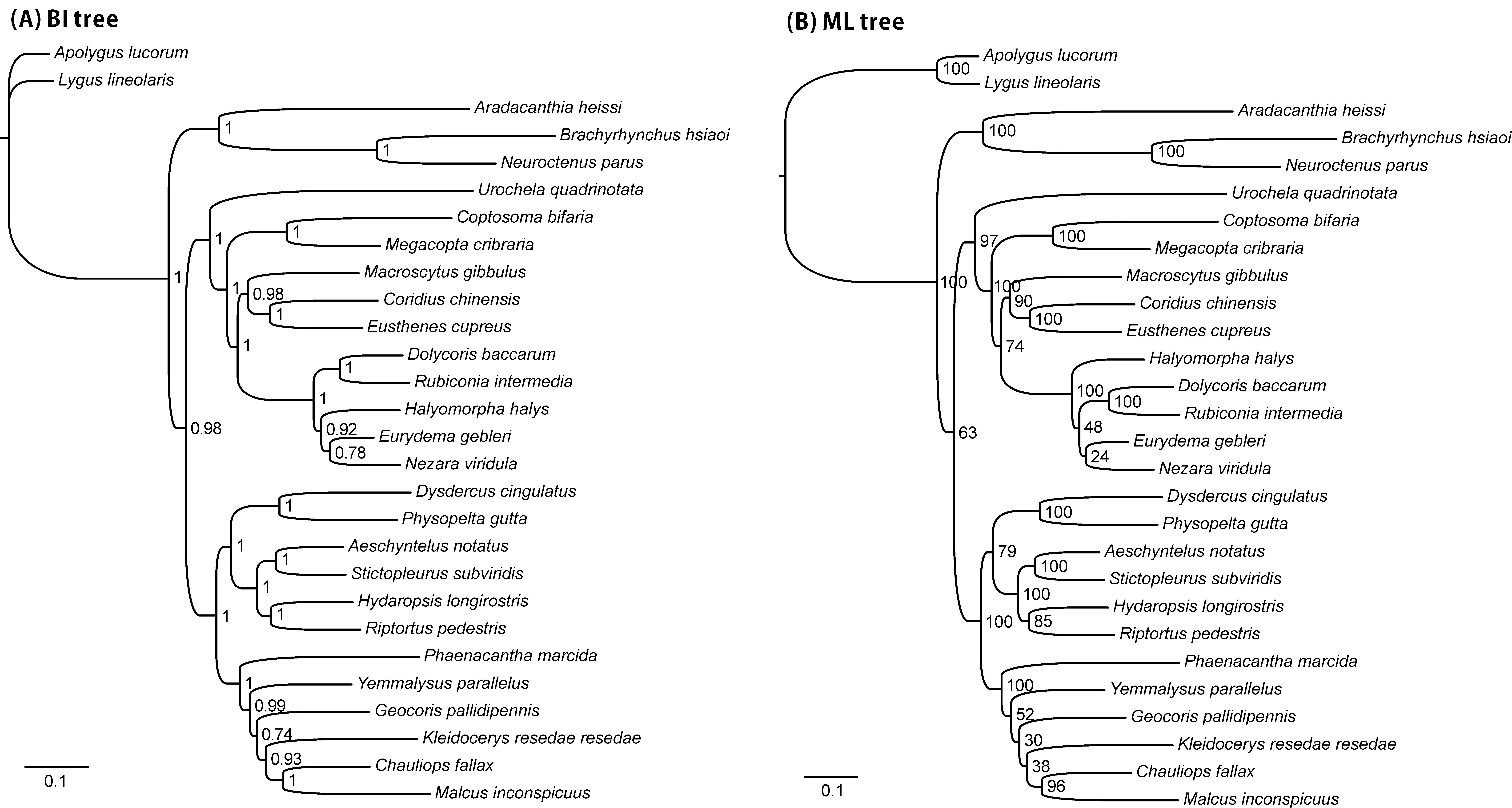

Supplement: Additional file 9: — Bayesian (A) and maximum likelihood (B) phylogeny of Pentatomomorpha inferred from the dataset P12. [file 12864_2015_1679_MOESM9_ESM.tiff]

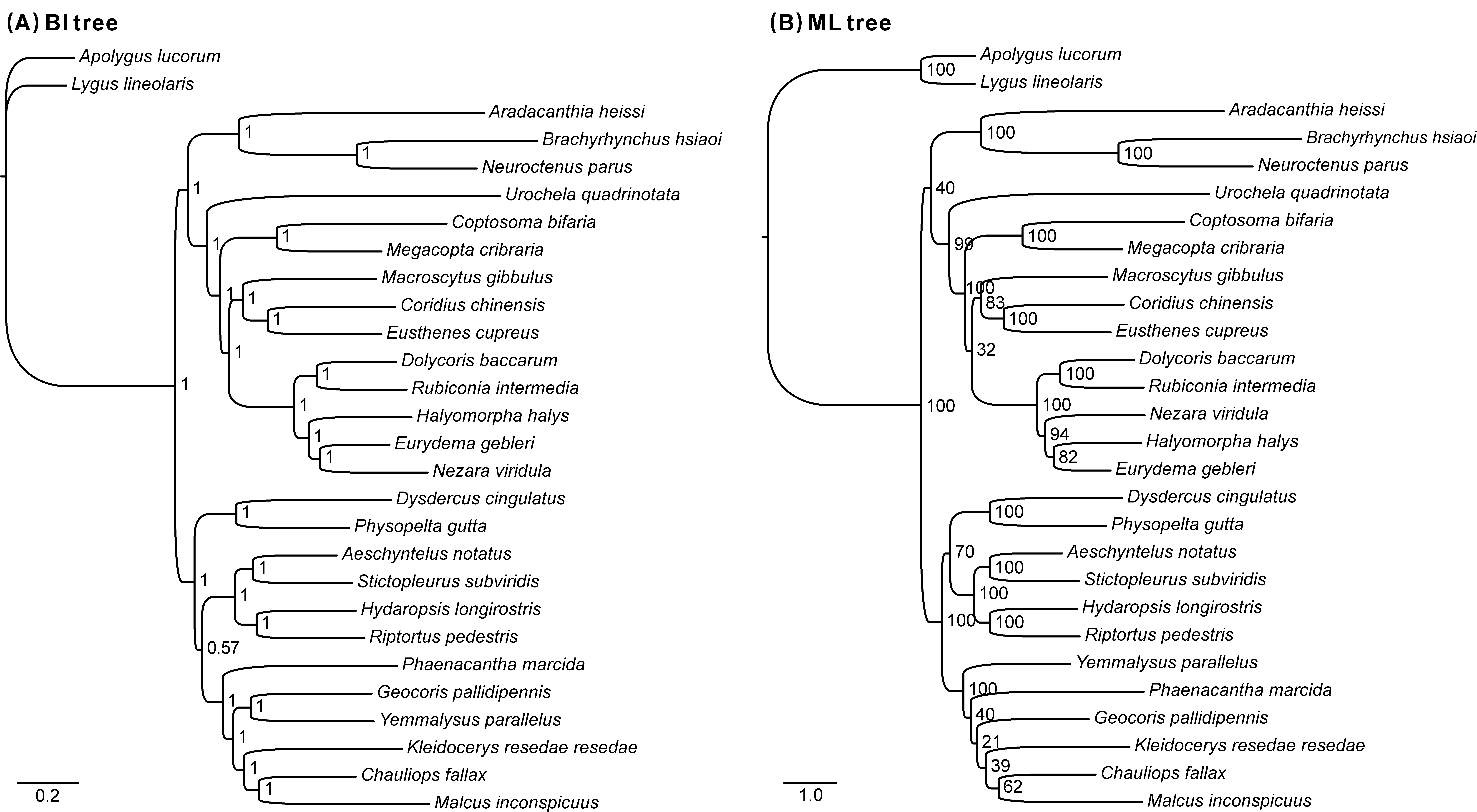

Supplement: Additional file 10: — Bayesian (A) and maximum likelihood (B) phylogeny of Pentatomomorpha inferred from the dataset P123RT. [file 12864_2015_1679_MOESM10_ESM.tiff]

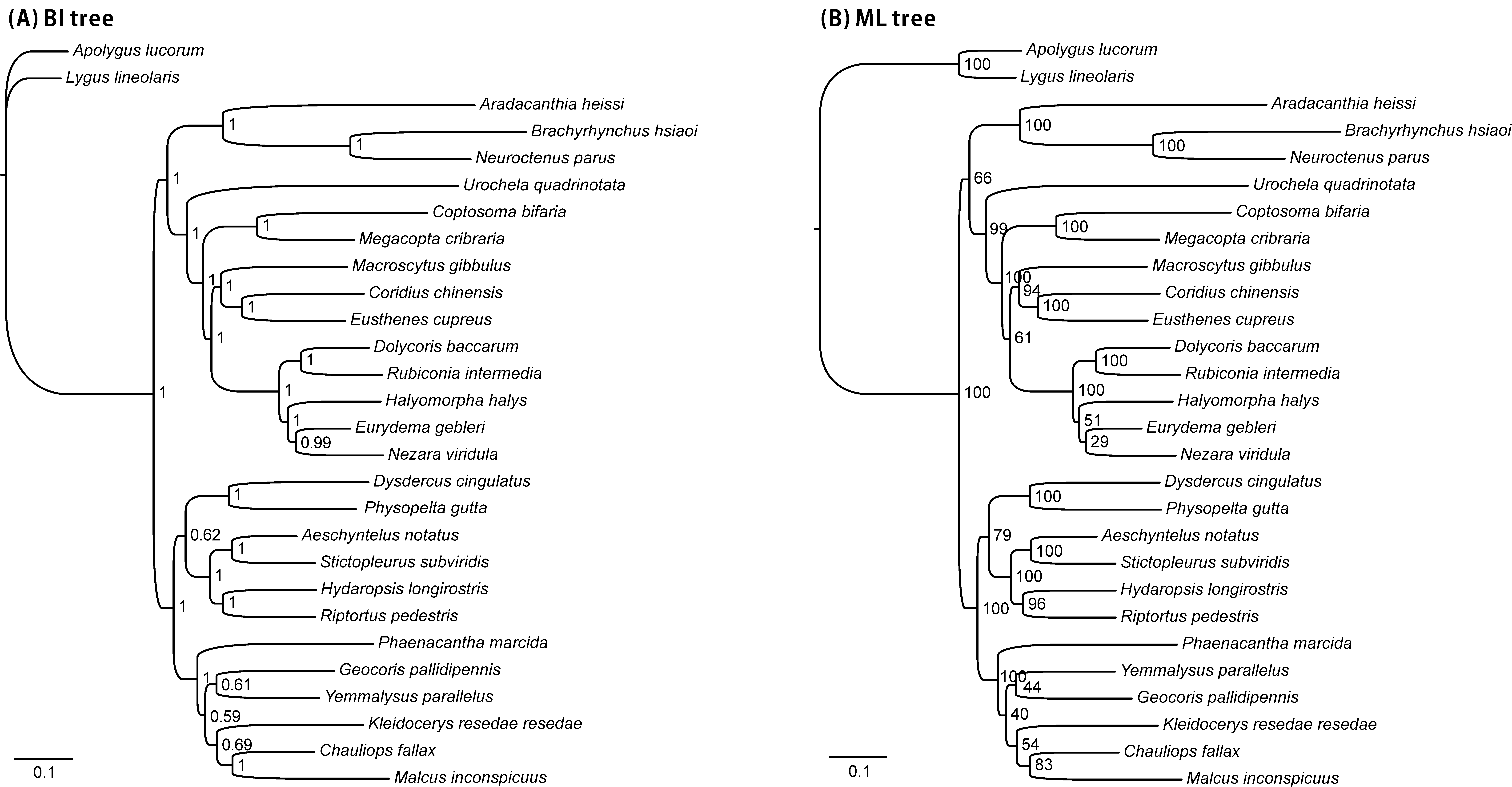

Supplement: Additional file 11: — Bayesian (A) and maximum likelihood (B) phylogeny of Pentatomomorpha inferred from the dataset P12LRT. [file 12864_2015_1679_MOESM11_ESM.tiff]

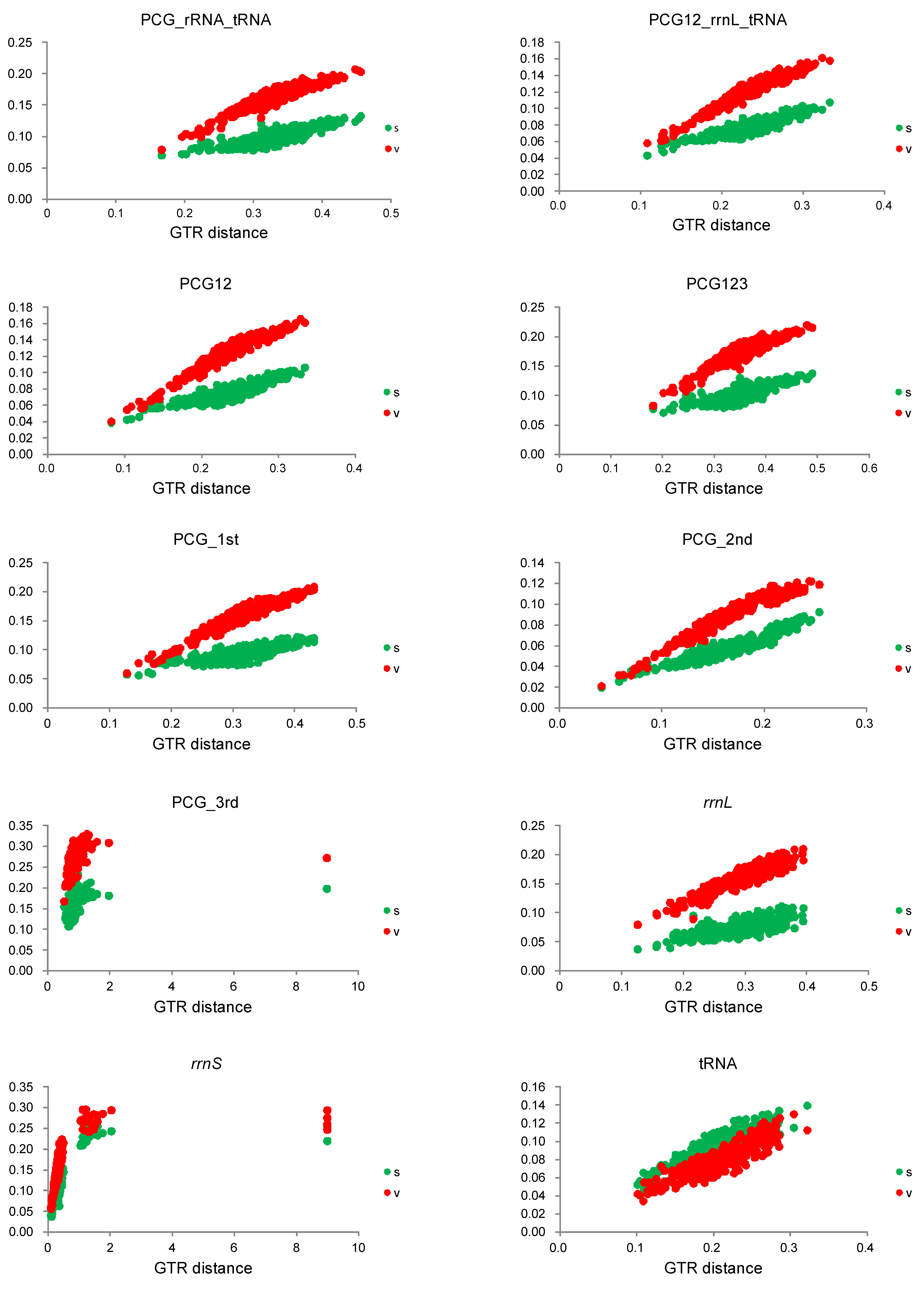

Supplement: Additional file 13: — Saturation plots for 13 protein-coding genes (PCGs), 2 rRNA genes ( rrnL and rrnS ) and 22 tRNA genes. [file 12864_2015_1679_MOESM13_ESM.tiff]
