# Supplementary material for: Comparative mitogenomic analysis of the superfamily Pentatomoidea (Insecta: Hemiptera: Heteroptera) and phylogenetic implications
Source: BMC Genomics. 2015 Jun 16;16(1):460. doi: 10.1186/s12864-015-1679-x (PMC4469028; doi:10.1186/s12864-015-1679-x)
Supplement: Additional file 14: — The best partitioning scheme selected by PartitionFinder for different datasets. [file 12864_2015_1679_MOESM14_ESM.docx]

**Additional file 14 The best partitioning scheme selected by PartitionFinder for the concatenated nucleotide sequences of 13 protein-coding genes**

| Dataset | Subset | Subset Partitions | Best Model |
| --- | --- | --- | --- |
| P123 | P1 | atp6_pos1, atp8_pos1, nad2_pos1, nad3_pos1, nad6_pos1 | GTR+I+G |
|  | P2 | atp6_pos2, atp8_pos2, cob_pos2, cox1_pos2, cox2_pos2, cox3_pos2, nad1_pos2, nad2_pos2, nad3_pos2, nad4L_pos2, nad4_pos2, nad5_pos2, nad6_pos2 | GTR+I+G |
|  | P3 | atp6_pos3, atp8_pos3, cob_pos3, cox1_pos3, cox2_pos3, cox3_pos3, nad2_pos3, nad3_pos3, nad6_pos3 | GTR+I+G |
|  | P4 | cob_pos1, cox1_pos1, cox2_pos1, cox3_pos1 | GTR+I+G |
|  | P5 | nad1_pos1, nad4L_pos1, nad4_pos1, nad5_pos1 | GTR+I+G |
|  | P6 | nad1_pos3, nad4L_pos3, nad4_pos3, nad5_pos3 | GTR+G |
| P12 | P1 | atp6_pos1, atp8_pos1, nad2_pos1, nad3_pos1, nad6_pos1 | GTR+I+G |
|  | P2 | cob_pos1, cox1_pos1, cox2_pos1, cox3_pos1 | GTR+I+G |
|  | P3 | nad1_pos1, nad4L_pos1, nad4_pos1, nad5_pos1 | GTR+I+G |
|  | P4 | atp6_pos2, atp8_pos2, cob_pos2, cox1_pos2, cox2_pos2, cox3_pos2, nad1_pos2, nad2_pos2, nad3_pos2, nad4L_pos2, nad4_pos2, nad5_pos2, nad6_pos2 | GTR+I+G |
| P123LRT | P1 | atp6_pos1, atp8_pos1, nad1_pos1, nad2_pos1, nad3_pos1, nad4L_pos1, nad4_pos1, nad5_pos1, nad6_pos1 | GTR+I+G |
|  | P2 | cob_pos1, cox1_pos1, cox2_pos1, cox3_pos1, tRNA | GTR+I+G |
|  | P3 | atp6_pos2, atp8_pos2, cob_pos2, cox1_pos2, cox2_pos2, cox3_pos2, nad1_pos2, nad2_pos2, nad3_pos2, nad4L_pos2, nad4_pos2, nad5_pos2, nad6_pos2 | GTR+I+G |
|  | P4 | rrnL | GTR+I+G |
| P123RT | P1 | atp6_pos1, atp8_pos1, nad2_pos1, nad3_pos1, nad6_pos1 | GTR+I+G |
|  | P2 | atp6_pos2, atp8_pos2, cob_pos2, cox1_pos2, cox2_pos2, cox3_pos2, nad1_pos2, nad2_pos2, nad3_pos2, nad4L_pos2, nad4_pos2, nad5_pos2, nad6_pos2 | GTR+I+G |
|  | P3 | atp6_pos3, atp8_pos3, cob_pos3, cox1_pos3, cox2_pos3, cox3_pos3, nad2_pos3, nad3_pos3, nad6_pos3 | GTR+I+G |
|  | P4 | cob_pos1, cox1_pos1, cox2_pos1, cox3_pos1, tRNA | GTR+I+G |
|  | P5 | nad1_pos1, nad4L_pos1, nad4_pos1, nad5_pos1, rrnL | GTR+I+G |
|  | P6 | nad1_pos3, nad4L_pos3, nad4_pos3, nad5_pos3 | GTR+G |
|  | P7 | rrnS | GTR+G |
